# Distressing psychotic-like experiences, cognitive functioning and early developmental markers in clinically referred young people aged 8–18 years

**DOI:** 10.1007/s00127-021-02168-9

**Published:** 2021-09-03

**Authors:** G. L. Barnes, C. Stewart, S. Browning, K. Bracegirdle, K. R. Laurens, K. Gin, C. Hirsch, C. Abbott, J. Onwumere, P. Banerjea, E. Kuipers, S. Jolley

**Affiliations:** 1 Department of Psychology, King’s College London, Institute of Psychiatry, Psychology and Neuroscience, London, SE5 8AF UK; 2 South London and Maudsley NHS Foundation Trust, London, SE5 8AZ UK; 3 NIHR Biomedical Research Centre (BRC) at the South London and Maudsley NHS Foundation Trust and Institute of Psychiatry, Psychology and Neuroscience, King’s College London, London, SE5 8AZ UK; 4 Department of Psychosis Studies, King’s College London, Institute of Psychiatry, Psychology and Neuroscience, London, SE5 8AF UK; 5 Queensland University of Technology (QUT), School of Psychology and Counselling, Brisbane, QLD 4059 Australia; 6 University of New South Wales, School of Psychiatry, Sydney, NSW 2052 Australia

**Keywords:** Childhood, Unusual experiences, Developmental milestone delays, Receptive language, Memory

## Abstract

**Purpose:**

Neurocognitive difficulties and early childhood speech/motor delays are well documented amongst older adolescents and young adults considered at risk for psychosis-spectrum diagnoses. We aimed to test associations between unusual or psychotic-like experiences (PLEs), co-occurring distress/emotional symptoms, current cognitive functioning and developmental delays/difficulties in young people (aged 8–18 years) referred to Child and Adolescent Mental Health Services in South London, UK.

**Methods:**

Study 1 examined receptive language, verbal learning and caregiver-reported speech and motor delays/difficulties in a sample of 101 clinically-referred children aged 8–14 years, comparing those reporting no PLEs (*n* = 19), PLEs without distress (*n* = 16), and PLEs with distress (*n* = 66). Study 2 tested associations of severity of distressing PLEs with vocabulary, perceptual reasoning, word reading and developmental delays/difficulties in a second sample of 122 adolescents aged 12–18 years with distressing PLEs.

**Results:**

In Study 1, children with distressing PLEs had lower receptive language and delayed recall and higher rates of developmental delays/difficulties than the no-PLE and non-distressing PLE groups (*F* values: 2.3–2.8; *p* values: < 0.005). Receptive language (*β* = 0.24, *p* = 0.03) and delayed recall (*β* = − 0.17, *p* = 0.02) predicted PLE distress severity. In Study 2, the cognitive-developmental variables did not significantly predict PLE distress severity (*β* values = 0.01–0.22, *p* values: > 0.05).

**Conclusion:**

Findings may be consistent with a cognitive-developmental model relating distressing PLEs in youth with difficulties in cognitive functioning. This highlights the potential utility of adjunctive cognitive strategies which target mechanisms associated with PLE distress. These could be included in cognitive-behavioural interventions offered prior to the development of an at-risk mental state in mental health, educational or public health settings.

## Introduction

There is now a rich literature on the processes involved in phenomena such as hearing or seeing things that others cannot, which emphasises the continuum of experience between healthy functioning and psychosis-spectrum diagnoses [1, 2]. Empirical research shows that such experiences are commonly reported by children and adolescents and are not always distressing or impairing [3], with mixed findings regarding their specificity for future psychosis [4–6]. However, these experiences are reliably associated with increased distress and risk of co-occurring non-psychotic mental health difficulties [7–9]. Investigating hypothesised factors driving associated distress in young people seeking help for mental health difficulties, therefore, has important implications for developing early detection and intervention efforts in clinical services.

Meta-analytic research has consistently identified a range of neurocognitive difficulties amongst adolescents and young adults who present with an identified at-risk mental state (ARMS) for psychosis, including lower pre-morbid IQ, working memory and attention difficulties [10, 11], and there is robust evidence implicating indicators of poor cognitive functioning in middle childhood as antecedents of schizophrenia-spectrum disorders [12].

However, considerably less is known about associations with cognitive functioning in youth referred to child mental health services with emotional and/or behavioural difficulties who also report unusual or psychotic-like experiences (PLEs). Terminology for such experiences, particularly in childhood, and in psychologically-oriented literature varies. Our group has previously written about ‘unusual experiences’ as young people participating in our studies advised that they preferred this term. However, acknowledging the potential scientific difficulties of generating multiple terms for related phenomena [13], we refer in this paper to PLEs for consistency with similar literature: our study materials retain the abbreviation UE(D), denoting unusual experiences (with distress). In our study, we delineate distressing PLEs as those that are either distressing/impairing in themselves and/or co-occur with general distress (i.e., emotional symptoms) [14].

There is some evidence for relationships between lower IQ, reading ability and working memory with PLEs in general population and hospital samples of children as young as 8 years of age and adolescents [15–18]. In recent prospective work [19, 20] in general population samples of youth, robust associations have been found between working memory and processing speed difficulties with PLEs, with some evidence that these difficulties persist into adulthood for young people who report PLEs, even when the PLEs are transient [20]. The general population literature seems to show a more robust link between domains of cognitive functioning and PLEs that are associated with impairment/distress [21] that will be important to investigate in other samples of youth.

Alongside neurocognitive functioning, early delays in language development and other developmental milestones have been found to distinguish those who later receive a psychosis-spectrum diagnoses from non-clinical controls [12]. Similar developmental markers are identified in samples of children and young adolescents reporting PLEs, including language processing difficulties [22], early speech and/or motor delays [23], and current fine motor skill difficulties [19, 20]. This literature therefore indicates that there may be subtle developmental lags among youth reporting PLEs and further investigation is now needed to test these associations in other groups, including young people reporting PLEs with associated impairment/distress.

Together, these findings seem to support the view that there is a cognitive-developmental pathway to PLEs [24], which has informed recommendations for cognitive behavioural interventions to be incorporated into clinical practice for youth reporting transient or persistent PLEs when they are associated with distress. For example, current UK Clinical Guidelines [25] recommend that interventions for distressing PLEs should employ strategies to help young people cope with distress, as well as techniques to improve general cognitive processes which may help to improve coping capacity in the future. However, more research is needed to identify which cognitive processes are most strongly implicated in PLE distress. This can inform recommendations for therapeutic targets within existing psychological interventions, both clinically and, potentially, in educational or public health settings. This may have important implications for considering the wider societal consequences for youth reporting distressing PLEs, particularly given growing evidence of associations between PLEs, poorer educational achievements [26–29] and later vocational status [30].

To date, limited research has investigated associations between distressing PLEs, cognitive functioning and developmental markers in transdiagnostic groups of young people referred to clinical child mental health services, which was the key aim of this study. In an initial study (Study 1), we aimed to investigate associations of PLE presence and distress with receptive language, verbal memory and parent-reported developmental delays/difficulties in a transdiagnostic sample of children aged 8–14 years referred to child mental health services for moderate to severe emotional and behavioural difficulties. In a second study (Study 2), we aimed to test these relationships in a larger transdiagnostic sample of adolescents aged 12–18 years reporting distressing PLEs, using standardised measures of vocabulary, perceptual reasoning and word reading.

## Method

### Participants

Study 1 participants were children aged between 8 and 14 years recruited for the Coping with Unusual Experiences Study [CUES; (ISRCTN:13766770)]. Study 2 participants were adolescents selected for self-reported distressing PLEs, recruited for the Coping with Unusual Experiences for 12–18-year-olds study [CUES + ; (ISRCTN: 21802136)].

The CUES and CUES + samples were composed of young people referred to community child and adolescent mental health services (CAMHS) within the South London & Maudsley (SLaM) NHS Trust, UK. SLaM covers four London boroughs, with high rates of ethnic diversity, population movement and socio-economic deprivation. Young people were referred for a range of mental health and behavioural difficulties and the samples were transdiagnostic. The CUES + group also included young people with diagnosed or emergent psychosis, as treated by the clinical teams.

For Study 1 and 2, exclusion criteria at screening and baseline were: insufficient English to complete the research assessments; and likely to move away from the local area within 6 months. For Study 1 only, youth were also excluded if they required specialist mental health or neurodevelopmental services. For Study 1, 110 young people and families contacted the research team and consented to participate. Of these, nine children were excluded as they did not complete the measures necessary for the current study (*n* = 5) or the family withdrew from the CUES study (*n* = 4). The remaining 101 children completed a measure of PLEs and cognitive functioning at baseline and were included in the study. For Study 2, 122 young people and their families (where appropriate) contacted the research team and consented to participate. All young people completed a measure of PLEs and cognitive functioning at baseline and were included in the current study.

### Study design and procedure

Ethical approval for both studies was obtained from the London-Hampstead Committee of the United Kingdom National Research Ethics Service (Study 1: Ref. 11/LO/0023; Study 2: Ref. 14/LO/1970).

For both studies, young people meeting inclusion criteria were invited by their local CAMHS team to find out more information regarding the study. For participants aged under 16 years, parental consent and child assent to contact with the research team was obtained. For those aged 16–18 years, the young person’s consent was obtained, and parental consent was sought with the young person’s agreement. This procedure was guided by the clinical team’s assessment of the young person’s capacity. Eligible young people (and families, where appropriate) were then sent an Information Sheet and Consent/Assent Forms, with a follow-up call from the research team within 2–4 weeks. Consenting young people and their families were then offered a baseline assessment with a researcher. Further details of the recruitment procedures are outlined in [14, 31].

### Measures

All participants in Study 1 and Study 2 completed the following measures:

### Psychotic-like experiences (PLEs)

The Unusual Experiences Questionnaire (UEQ; also known as the Psychotic-Like Experiences Questionnaire for Children–PLEQ-C [23]) is a self-report measure which assesses unusual ideas and perceptions in young people. The measure was initially designed and tested for use with children aged 9–12 years, however, the measure has since been developed and validated for use in older adolescent samples [32] and recent studies have used a wider age range of young people aged 7–17 years [7, 14, 31]. Briefly, the UEQ asks young people to endorse nine different PLEs which are rated on a 3-point scale for *Conviction*: 0 (not true); 1 (somewhat true); 2 (certainly true); *Frequency* over the past 2 weeks: 0 (not at all); 1 (only once); 2 (2–4 times); 3 (5 + times); *Distress* (‘How much has it upset you?’) and functional *Impact* (‘How much has it made things hard at home or school?’), both rated: 0 (not at all); 1 (only a little); 2 (quite a lot); 3 (a great deal). Item totals (ratings across *Conviction, Frequency, Distress* and *Impact*) are summed to create a severity score for each endorsed PLE (range = 0–11) and a PLE distress severity score is calculated by selecting items where distress/impact (> 0) is endorsed (range = 1–99). The UEQ has established criterion validity against clinical interview and predictive validity for the persistence of PLEs into adolescence [33]. Young people in Study 1 were unselected with respect to PLEs. For Study 2, young people needed to endorse at least one PLE, rated >  = 1 on distress or adverse impact, or accompanied by a score in the clinical range (> 6) on the SDQ-ES scale (see below).

### General psychopathology

The Strengths and Difficulties Questionnaire (SDQ [34]) is a self-report questionnaire assessing emotional and behavioural difficulties in young people aged 8–17 years. There are five subscales assessing Emotional Symptoms, Peer Relationship Problems, Hyperactivity/Inattention, Conduct Problems and Prosocial Behaviours. Each scale has five items rated on a 0–5 scale; with higher scores indicating greater difficulty, except for the Prosocial scale. Subscale scores (0–10) for Emotional Symptoms, Peer Relationship Problems, Hyperactivity/Inattention and Conduct Problems are summed to create a Total Difficulties score (0–40). The SDQ has robust psychometrics in child and adolescent samples [31]. For this study, we used the composite Total Difficulties score as an index of global psychopathology severity. In Study 2, two methods of defining distressing PLEs were employed: a PLE accompanied by a score in the clinical range of the Emotional Symptoms subscale (> 6); and/or a PLE self-rated for distress/adverse impact.

### Speech and motor development

A brief caregiver report [23] was used to assess early developmental delays or difficulties in speech and motor functioning. The measure is comprised of nine items which ask caregivers about the ages at which their child achieved their key speech/motor milestones (three items), parent/professional concerns regarding early delays or difficulties in speech or motor development (five items), and current difficulties in motor functioning (i.e. co-ordination problems) (one item). Speech delays/difficulties are coded if a speech delay or parent/professional concern prior to aged 3 years is reported (0—no speech delay/concern prior to 3 years, 1—at least one speech delay/concern prior to 3 years). Motor delays/difficulties are coded if a motor delay or a parent/professional concern prior to age 3 years is reported, or if parents report a current difficulty with motor functioning (0—no motor delay/concern; 1—at least one motor delay/concern). In the present studies, a composite variable is used to capture the presence of any speech/motor delay or difficulty (0—no developmental delay/difficulty; 1—≥ 1 developmental delay/difficulty).

### Neurocognitive performance

For Study 1, participants completed the following cognitive assessment measures:

#### Receptive language

The British Picture Vocabulary Scale (BPVS II [35] is a standardised assessment of receptive vocabulary acquisition in children aged 3–15 years. Participants are shown four grid drawings and asked to name the image which corresponds to a word spoken by an administrator. The sum of correct answers forms the raw score, which is calculated into a standardised score based on age and sex. For this study, standardised BPVS scores and their qualitative ranges are reported (Below Average =  < 90; Average = 90–110; Above Average =  > 110).

#### Verbal learning

The Rey Auditory Verbal Learning Test (RAVLT [36]) is an assessment of verbal language ability. Administration followed a standardised adaptation for 7–15-year-olds [37]. An initial list of 15 words (List A) is read aloud five times, with free recall after every presentation (Trials I–V). A second distractor list of 15 unrelated words (List B) is read aloud and recalled once; then participants recall List A immediately afterward, and again after a 21-min delay. Participants are then presented with a list of 30 words (words from Lists A and B, and new words not present on either) which forms a recognition task. The RAVLT consists of five indices of verbal language ability: (1) *Immediate Recall* (Trial I score); (2) *Acquisition* (sum of Trial I–V); (3) *Delayed Recall;* (4) *Recognition*; and (5) *False Recognition.* For this study, standardised RAVLT scores and their qualitative ratings (based on the number of standard deviations from the mean) are reported (Below Average =  > − 1.5 SD; Average =  ±  0–1 SD; Above Average =  <  + 1.5 SD).

For Study 2, participants completed the following cognitive assessment measures:

#### Cognitive functioning

The Wechsler Abbreviated Scale of Intelligence—2nd Edition (WASI-II [38]) is a standardised measure of cognitive functioning in individuals aged 6–89 years. It has four sub-tests: Vocabulary; Block Design; Similarities and Matrix Reasoning. The Vocabulary and Matrix Reasoning sub-tests are combined into a FSIQ-2 score, standardised by age, which can be used as an estimate of general cognitive ability. For this study, FSIQ-2 Scores were calculated and reported for descriptive purposes only. Standardised scaled scores for the Vocabulary and Matrix Reasoning sub-tests were calculated and categorised into qualitative ranges (Below Average =  < 7, Average = 8–12; Above Average =  ≥ 13) and were included in the analysis.

#### Word reading

The Wechsler Individual Achievement Test–Second edition (WIAT-II [39]) is a standardised, comprehensive measure of reading, language and numerical attainment in young people aged 4–25 years. It consists of 16 subtests assessing specific attainment domains, which are standardised according to age and sex. For our study, standardised Word Reading scores were used as an index of current word reading ability and categorised into qualitative ranges (Below Average =  < 90; Average = 90–110; Above Average =  > 110).

### Statistical analysis

Analyses were performed using the Statistical Package for the Social Sciences, Version 25 (SPSS 25) [40].

In Study 1, young people were categorised into three groups: A ‘No-PLE’ group, comprising those who endorsed no PLEs (Conviction scores all = 0 on the UEQ); a ‘PLE’ group, comprised of those reporting ≥ 1 PLE (Conviction score > 0) but rating no Distress on the UEQ (Distress/Impact score = 0); and a ‘distressing PLE’ group, comprised of children endorsing ≥ 1 PLE and distress (conviction and distress/ Impact score > 0).

In an initial analysis step investigating bivariate associations, Chi square tests were performed with PLE group (0 = no PLE; 1 = PLE; 2 = distressing PLE) as the independent variable. Dependent factors comprised: the categorical BPVS variable, the five RAVLT indices (all 0—below average; 1—average; 2—above average), and the developmental delay/difficulty variable (0—no delay/difficulty; 1—at least 1—delay/difficulty). The three groups were compared by age, sex and ethnicity.

Total scores for each measure were prorated if ≤ 2 items were missing. Initial data inspection indicated that multicollinearity between the dependent variables was within acceptable limits (VIFs ≤ 2.2; tolerance values > 0.4). Inspection of the standardised residuals indicated non-normal distributions for RAVLT acquisition, recognition, and false recognition, confirmed by the calculation of z-scores for skewness and kurtosis (z skewness/standard error (SE) skewness; kurtosis/SE kurtosis) > 1.96). Inspection of outliers showed that all values for RAVLT acquisition lay within three standard deviations (SDs) of the mean; but not for recognition and false recognition. A natural log transform was applied to these two variables (value = ln (value + 1)), rendering all values within three SDs of the mean.

To investigate associations of cognitive functioning and developmental delays/difficulties with PLE distress, the distressing PLE group (*n* = 66) was included in a linear regression analysis, with PLE distress severity as the dependent variable. Variables found to be significantly associated with PLE group membership in the Chi-squared analyses were entered as predictors, controlling for sex and general psychopathology (SDQ Total Difficulties). Multicollinearity remained within acceptable limits (VIFs < 2.3; tolerance > 0.4), and inspection of standardised residuals confirmed that the assumption of normality was unviolated (KS statistic = 0.1, d.f. = 52, *p* = 0.2), so no transformation was applied to the data.

In Study 2, variables were first examined for skewness and kurtosis; Kolmogorov–Smirnov (K–S) tests were used to check for normality. Inspection of values indicated that severity of distressing PLEs (skewness = 1.06; kurtosis = 1.08) was the only variable that was non-normally distributed. Further inspection of the standardised residuals indicated non-normal distributions for severity of distressing PLEs (KS = 0.101, *p* = 0.004) and WIAT word reading (KS = 0.377, *p* = 0.002), though all outliers lay within 2 SDs of the mean. As these variables were non-normally distributed, non-parametric tests were performed.

In an initial analysis step, correlational analyses (or Chi-squared tests for categorical variables) were performed to investigate associations between PLE distress severity, the neurocognitive variables (Vocabulary and Matrix Reasoning scaled scores), the composite developmental delay/difficulty variable (0—no delay/difficulty; 1—at least 1 delay/difficulty), sex and ethnicity.

Next, multiple regression analysis was conducted, using the ‘Enter’ method, to investigate associations of Vocabulary, Matrix (Perceptual) Reasoning, Word Reading and speech and motor delays/difficulties with PLE distress severity, which was entered as the dependent variable. In Block 1, sex and SDQ Total Difficulties score were entered to control for their effect on PLE distress severity. In Block 2, Vocabulary, Matrix Reasoning and Word Reading standardised scores and the dichotomous developmental delay/difficulty variable. were entered as hypothesised predictors of PLE distress severity in the sample. Multicollinearity between the variables was within acceptable limits (VIF values all > 1.0; tolerance values > 0.6) and inspection of the standardised residuals confirmed that the assumption of normality was unviolated (KS statistic = 0.08, *d.f.* = 122, *p* = 0.51), so no transformation was applied to the data.

A power analysis was conducted using the G-Power programme [41] to calculate the achieved power for multiple regression analyses with the sample sizes obtained. This identified that the minimum sample size needed to identify a small effect size (*β* = 0.15) was *n* = 55. Therefore, for Study 1, we estimated that a sample size of *n* = 66 (the distressing PLE group) and for Study 2, a sample size of *n* = 122 would have 80% power to detect a significant small effect of the independent variables on PLE distress severity in the multiple regression analyses.

## Study 1 results

### Baseline characteristics

A total of 101 young people participated in the study. Participants had a mean age of 11.6 years (SD = 2.0) and the majority were male (60%). The most commonly represented ethnicity was White British/Other (49.5%), followed by Black British/African/Caribbean (23.8%) and Dual Heritage (15.8%). Almost all the sample were enrolled in full-time education (99%); just one participant was not in education. Demographic and clinical characteristics of the sample are outlined in Table 1.

**Table 1:** Demographic and clinical characteristics of participants in Studies 1 and 2

	Study 1 (*n* = 101)	Study 2 (*n* = 122)
Age (years), M (SD)	11.6 (2.0)	14.8 (1.6)
Gender, *n* (%)		
Male	61 (60.0)	29 (23.8)
Female	40 (40.0)	93 (76.2)
Ethnicity, *n* (%)		
White British/other	50 (49.5)	57 (46.7)
Black British/African/Caribbean	24 (23.8)	25 (20.5)
Asian British/other	6 (5.9)	10 (8.2)
Dual heritage	16 (15.8)	24 (19.7)
Other	5 (5.0)	6 (4.9)
Educational status		
Full time education or work	100 (99.0)	108 (88.5)
Home schooled	0 (0.0)	2 (1.6)
In hospital	0 (0.0)	4 (3.3)
Not in education, employment or training	1 (1.0)	8 (6.6)
SDQ Total, *M* (SD)	16.8 (6.7)	20.5 (5.5)

*M* mean, *SD* standard deviation, *n* number of participants, *SDQ* Strengths and Difficulties Questionnaire (Goodman, 2001)

### Rates of PLEs in the sample

In total, 81% of the sample (*n* = 82) reported a recent PLE (past 2 weeks); of whom 20% did not report distress (PLE group *n* = 16); and 80% did (distressing PLE *n* = 66). All but one participant in the distressing PLE group reported associated distress/adverse impact on the UEQ. This young person reported > 1 PLE and co-occurring emotional symptoms on the SDQ that fell within the clinical range (> 6). Overall, 19% of the sample did not report a recent PLE (no PLE group *n* = 19). There were no significant differences between the groups in terms of sex (*χ*^2^ = 0.43, *p* = 0.12), ethnicity (*χ*^2^ = 0.46, p = 0.80), or age (*F* (2, 98) = 0.63; *p* = 0.54). Young people self-reported moderate to severe total difficulties on the SDQ, with the highest levels in the distressing PLE group (*F* (2, 93) = 14.3; *p* < 0.001).

*Associations between cognitive performance and PLEs.*

In total, 101 young people completed the BPVS and 97 completed the RAVLT. Qualitative ranges for standardised scores across the groups are outlined in Table 2.

**Table 2:** Scores on each dependent variable for participants in Study 1 (*n* = 101) and tests of bivariate associations with group

	No PLE (*n* = 19)		PLE no distress (*n* = 16)		Distressing PLE (*n* = 66)		TOTAL (*n* = 101)		*χ*^2^	*p*
	*M*/*n*	SD/%	*M*/*n*	SD/%	*M*/*n*	SD/%	*M*/*n*	SD/%		
BPVS, *M* (SD)	95.4	16.1	93.1	10.1	88.9	16.7	90.8	15.9	**9.9**	0.04*
Below average [56–89]	4	(21.2)	5	(31.3)	35	(53.0)	44	(43.6)		
Average [90–109]	10	(52.6)	10	(62.5)	22	(34.8)	43	(42.6)		
Above average [110–132]	5	(26.3)	1	(6.3)	9	(12.1)	14	(13.9)		
RAVLT^a^										
Immediate recall (Trial I), M (SD)	5.7	1.9	5.4	1.6	5.3	1.9	5.4	1.9		
Below average [− 1.5 SD]	8	(42.1)	4	(26.7)	22	(35.5)	34	(35.4)	1.9	0.80
Average [0–1 SD]	10	(52.6)	11	(73.3)	37	(59.7)	58	(60.4)		
Above average [+ 1.5 SD]	1	(5.3)	0	(0.0)	3	(4.8)	4	(4.2)		
Acquisition (Trial I–V), M (SD)	46.1	(11.0)	45.0	(6.4)	40.0	(10.9)	42.0	(10.6)		
Below average [− 1.5 SD]	7	(36.8)	7	(43.8)	40	(64.5)	54	(55.7)	7.8	0.09
Average [0–1 SD]	10	(52.6)	9	(56.3)	18	(29.0)	37	(38.1)		
Above average [+ 1.5 SD]	2	(10.5)	0	(0.0)	4	(6.5)	6	(6.2)		
Delayed recall, *M* (SD)	10.0	(2.4)	8.4	(3.2)	8.1	(3.3)	8.6	(3.2)		
Below average [− 1.5 SD]	5	(26.3)	6	(37.5)	37	(59.7)	48	(49.5)	**9.6**	0.04*
Average [0–1 SD]	12	(63.2)	10	(62.5)	21	(33.9)	43	(44.3)		
Above average [+ 1.5 SD]	2	(10.5)	0	(0.0)	4	(6.4)	6	(6.2)		
Recognition, *M* (SD)	13.5	(2.0)	13.7	(1.4)	12.7	(3.2)	13.0	(2.8)		
Below average [− 1.5 SD]	5	(26.3)	3	(18.8)	16	(26.2)	24	(25.0)	0.69	0.95
Average [0–1 SD]	13	(68.4)	12	(75.0)	43	(70.5)	68	(70.8)		
Above average [+ 1.5 SD]	1	(5.3)	1	(6.3)	2	(3.3)	4	(4.2)		
False recognition	1.0	(1.2)	2.1	(3.6)	1.9	(2.8)	1.8	(2.7)		
False positives < 2	16	(84.2)	11	(68.8)	36	(59.0)	63	(65.6)	4.2	0.13
False positives ≥ 2	3	(15.8)	5	(31.2)	25	(41.0)	33	(34.4)		
Developmental delay/difficulty^b^	N	%	N	%	N	%	N	%	**8.4**	0.01*
Yes	0	(0.0)	2	(10.5)	17	(37.0)	19	(26.8)		
No	16	(100)	7	(89.5)	29	(63.0)	52	(73.2)		

Note: *PLE* psychotic-like experiences, *BPVS* British Picture Vocabulary Scale (Dunn et al. 1997); *RAVLT* Rey Auditory Verbal Learning Test (Schmidt, 1996); M = Mean, *SD* standard deviation

^a^Total *N* = 97 (distressing PLE group *n* = 62)

^b^Total *n* = 71 (no PLE *n* = 16; PLE no distress *n* = 9; distressing PLE *n* = 46)

Significant between-groups differences were found for receptive language ability (BPVS standardised scores): *χ*^2^ = 9.9, *p* = 0.04. Inspection of the adjusted residuals indicated that, compared to the no PLE group, a significantly greater proportion of children in the distressing PLE group (adjusted residual = 2.1) performed in the ‘Below Average’ range for receptive language than would be expected by chance.

We found significant between-groups differences for the delayed recall subtest of the RAVLT; *χ*^2^ = 9.6, *p* = 0.04. Inspection of the adjusted residuals indicated that, compared to the no PLE group, a significantly greater proportion of children in the distressing PLE group (adjusted residual = 2.1) performed in the ‘Below Average’ range for delayed recall. There was evidence for an effect of group membership on the acquisition (total verbal learning) trial of the RAVLT that did not reach significance at the 5% level: *χ*^2^ = 7.8, *p* = 0.09. There were no significant group differences on RAVLT indices of immediate recall (*χ*^2^ = 1.9, *p* = 0.80), recognition (*χ*^2^ = 0.69, *p* = 0.95) or false recognition (*χ*^2^ = 4.2, *p* = 0.13).

### Associations between speech/motor delays or difficulties and PLEs

Caregiver-reported developmental milestone data were available for 71 participants (70% of the sample). Rates of speech/motor delays or current difficulties across the three groups are reported in Table 2. Overall, 73% (*n* = 52) of caregivers who completed the measure did not retrospectively report a developmental delay or current difficulty. Rates of speech/motor delays or difficulties were lowest in the no-PLE group (*n* = 0, 0%) and highest in the distressing PLE group (*n* = 17, 37%). There were also low rates in the non-distressing PLE group (*n* = 2, 10.5%). Young people with distressing PLEs were significantly more likely to have early developmental delays or a current difficulty compared to those with no PLEs: *χ*^2^ = 8.4, *p* = 0.01 (adjusted residual = 2.0).

### Associations of cognition and development with severity of distressing PLEs

When severity of distressing PLEs was predicted from the cognitive and developmental variables, receptive language ability (*β* = 0.24, *p* = 0.03) and delayed recall (*β* = -0.17, *p* = 0.02) were significant independent predictors of severity of distressing PLEs when controlling for demographic variables and general psychopathology. Early speech/motor delays or current difficulties was not a significant predictor of severity of distressing PLEs (*β* = 0.23, *p* = 0.08). The regression model statistically predicted total severity of distressing PLEs, *F* (4, 61) = 3.2, *p* = 0.02, accounting for 17% of the variance. The final regression model is outlined in Table 4.

## Study 2 results

### Baseline characteristics

A total of 122 young people participated in the study. Participants had a mean age of 14.8 years (SD = 1.6) and the majority were female (76.2%). The most commonly represented ethnicity was White British/Other (46.7%), followed by Black British/African/Caribbean (20.5%) and Dual Heritage (19.7%). Most young people (88.5%) were enrolled in full-time education or work; the rest were home schooled (1.6%), in hospital (3.3%) or not currently enrolled in education, training or work (6.6%). All but one participant reported associated PLE distress/adverse impact on the UEQ. This young person reported > 1 PLE and co-occurring emotional symptoms on the SDQ which fell within the clinical range (> 6). Demographic characteristics of the sample are outlined in Table 1.

### Associations of cognitive-developmental functioning with PLE distress severity

All participants completed the WASI-II and WIAT-II Word Reading sub-test, and 85 (70%) caregivers completed the speech/motor functioning measure. Mean standardised scores and frequencies (qualitative ranges) for the variables are reported in Table 3.

**Table 3:** Mean scores on all variables for participants in Study 2 (*n* = 122)

	*M*/*n*	(SD)/%
WASI-II		
FSIQ Score, *M* (SD)	100.3	(16.1)
Below average [range 56–89]	36	29.5
Average [range 90–109]	59	48.4
Above average [range 110–132]	27	22.1
Vocabulary, *M* (SD)	10.2	(3.3)
Below average [range 1–7]	25	20.5
Average [range 8–12]	65	53.3
Above average [13–19]	32	26.2
Matrix reasoning, M (SD)	9.2	(3.5)
Below average [range 1–7]	38	31.1
Average [range 8–12]	65	53.3
Above average [13–19]	19	15.6
WIAT-II		
Word reading, *M* (SD)	103.1	(13.8)
Below average [range 66–89]	15	12.3
Average [range [range 90–109]	61	50.0
Above average [range 110–134]	46	37.7
Any developmental delay/difficulty^a^		
Yes	36	(42.4)
No	49	(57.6)

*WASI-II* Weschler Abbreviated Scale of Intelligence-Second Edition (Wechsler, 2011), *FSIQ* Full Scale IQ, *WIAT-II* Weschler Individual Achievement Test-Second Edition (Wechsler, 2001), *M*  mean, *SD* standard deviation

^a^Total *n* = 85

There were no significant associations between ethnicity and the neurocognitive variables: vocabulary (*F* (26, 95) = 1.24, *p* = 0.23); matrix reasoning (F (22, 99) = 0.91, *p* = 0.59); word reading (*F* (1, 63) = 1.29, *p* = 0.18), or caregiver-reported developmental delays/difficulties (*F* (1, 83) = 0.01, *p* = 0.92), Sex was significantly associated with early speech/motor delays or current difficulties (χ^2^ = 5.8, *p* = 0.02). Inspection of the adjusted residuals indicated that a higher proportion of boys had an early developmental delay or current speech/motor difficulty compared to girls (adjusted residual = 2.3). General psychopathology (SDQ Total Difficulties) was significantly correlated with Word Reading (r = −0.19, *p* = 0.04) so this was controlled for in the multivariate analyses. In the final regression models, the cognitive-developmental variables did not significantly predict the severity of distressing PLEs when controlling for sex and general psychopathology: vocabulary (*β* = 0.17, *p* = 0.18); matrix reasoning (*β* = -0.22, *p* = 0.09), word reading (*β* = −0.01, *p* = 0.91); speech/motor delays or current difficulties (*β* = 0.13, *p* = 0.25). General psychopathology (SDQ total score) was a significant predictor of severity of distressing PLEs in the model (*β* = 0.37, *p* = 0.001). The regression model statistically predicted total severity of distressing PLEs, *F* (7, 77) = 2.91, *p* = 0.009, accounting for 20.9% of the variance. The final regression model is outlined in Table 4.

**Table 4:** Step-wise regressions of dependent variables with severity of distressing PLEs for Study 1 and Study 2

Independent variable	Uncontrolled *β*	Controlled *β*^1^	95% CI for *β*^1^	*p* for *β*^1^
Study 1 (*n* = 66)				
BPVS standardised Score	0.31	0.24	0.78–13.52	0.03*
RAVLT delayed recall	− 0.28	− 0.17	− 14.44 to 1.24	0.02*
Any developmental delay/difficulty	0.26	0.23	− 1.28 to 22.12	0.08
Study 2 (*n* = 122)				
WASI-II Vocabulary	0.19	0.17	− 0.37 to 1.98	0.18
WASI-II Matrix Reasoning	− 0.17	-0.22	− 1.97 to 0.16	0.09
WIAT-II Word Reading	− 0.09	− 0.01	− 0.50 to 0.45	0.91
Any developmental delay/difficulty	0.10	0.13	− 2.97 to 11.25	0.25

*PLE* psychotic-like experiences, *BPVS* British Picture Vocabulary Scale (Dunn et al. 1997), *RAVLT* Rey Auditory Verbal Learning Test (Schmidt et al. 1996), *WASI-II* Weschler Abbreviated Scale of Intelligence-Second Edition (Wechsler, 2011), *WIAT-II* Weschler Individual Achievement Test-Second Edition (Weschler, 2001), *n* number of participants

*β*^1^ Standardised beta coefficient, controlling for sex and general psychopathology

## Discussion

Using samples of young people already referred to child and adolescent mental health services (cf. specific at-risk services or from the general population), we aimed to investigate relationships between PLEs, associated distress, cognitive functioning and early developmental delays/difficulties in a sample of children aged 8–14 years, and in a second sample of adolescents aged 12–18 years reporting distressing PLEs. The results of Study 1 showed associations between receptive language and verbal memory with PLE presence and distress severity, even after controlling for demographic variables and general psychopathology. In Study 2, we did not find significant associations between cognitive functioning and the severity of distressing PLEs.

The results of Study 1 are potentially consistent with a cognitive-developmental model of PLEs [24], in that PLEs are associated with subtle difficulties in specific domains of cognitive functioning, indistinguishable from, but at the lower end of, average performance, while distressing PLEs are associated with more severe cognitive difficulties, distinguishable from average performance, and increasing with greater PLE distress severity. These findings build on existing research from the general population [15–18] and in adolescents and young adults who present with an identified at-risk mental state (ARMS) for psychosis [10–12].

In terms of hypothesised cognitive processes, problems with information retention (evidenced by lower delayed recall in Study 1) may arise from context processing difficulties, increasing proneness to anomalous experiences. In addition, difficulties with receptive language (Study 1) may impact on a young person’s ability to understand information presented to them verbally and use verbal reasoning to define concepts, which could lead to unhelpful appraisals of anomalous experiences, and both of these factors are hypothesised to maintain distress in cognitive models of psychosis [42]. Difficulties with information retention and expressive/receptive language may also play a role in cognitive biases, such as jumping to conclusions, as is found in samples of young adults with first episode psychosis [43]. In relation to the current study, it could be that children whose PLEs are not distressing are better cognitively equipped to identify adaptive appraisals for their experiences, whereas, children with distressing PLEs may have reduced capacity to evaluate and form adaptive explanations of these experiences. However, it is unclear whether distressing PLEs have a different cognitive effect than non-distressing PLEs, or whether distress and PLEs impact separate cognitive processes, which requires further investigation in methodologically robust studies.

Speech/motor developmental delays are more likely among male than female youth in the general population [44] and in both Study 1 and Study 2, we found that male children reporting distressing PLEs were significantly more likely to have a parent-reported developmental delay or current difficulty than female youth. While these developmental delays did not statistically predict the severity of distressing PLEs in either sample, youth with distressing PLEs were significantly more likely than those without PLEs to experience delays (Study 1). This finding is potentially consistent with a sex-by-developmental delay interaction effect on distressing PLEs that may reflect an early neurodevelopmental vulnerability model in male youth [45]. It should be noted that the Study 2 sample was comprised predominantly of female adolescents reporting distressing PLEs and more severe general psychopathology, which could indicate sampling bias. In a recent study [7], we found that for young people referred to child and adolescent mental health services who completed the UEQ at initial screening (85%), only one third of males compared to two thirds of females reported distressing PLEs, which highlights potential sex variation in referral patterns and research/treatment access for young people reporting PLEs. However, the fact that we did not find associations between cognitive-developmental markers and severity of distressing PLEs in this sample possibly indicates a later mood and emotional dysregulation model of distressing PLEs in females, which is consistent with previous research [8, 46]. This means that associations of distressing PLEs with specific aspects of cognitive and developmental functioning may become less pronounced with age and vary according to sex, with other emotional, cognitive and behavioural processes becoming more pertinent in distress maintenance over time. However, these hypotheses are tentative given the limitations of the study designs, and more robust prospective investigation is needed to explore associations further.

Study limitations should be noted. All participants were young people referred to community CAMHS and we did not recruit non-clinical control groups. Therefore, findings may not generalise to general population samples nor to samples with an identified ARMS for psychosis, though some young people may have met criteria. In addition, we adopted our inclusion criteria to fit our target population and relied on routine clinical assessment to judge severity of mental health difficulties, without conducting our own formal diagnostic assessments at baseline. Moreover, inclusion criteria for the two studies differed; in Study 2, only participants with distressing PLEs were included, and we did not recruit a no distress group, so between-group analyses were not possible. In addition, in Study 1, both the no-PLE and PLE groups had a small number of participants which may limit the statistical power of the study to test the comparisons reported.

Another limitation is that we did not use the same cognitive assessment measures and assessed different areas of cognition in the two studies which limits the comparisons that can be made across the samples. Finally, speech and motor functioning were assessed using a retrospective caregiver report, rather than more robust prospective measures of developmental milestones. These study limitations, alongside the cross sectional designs, means that claims cannot be made about whether there is a causal relationship between cognitive-developmental functioning and distressing PLEs.

Further investigation using longitudinal study designs and multivariate approaches should now seek to investigate associations between distressing PLEs and cognitive-developmental markers in samples of youth. This could include a combination of prospective and retrospective measures and incorporate other cognitive domains (e.g. IQ, working memory and processing speed) shown to be associated with PLEs in the general population [15–18] and among young people with an ARMS for psychosis [10–12]. This may help to disentangle associations between specific developmental markers and PLEs in young people and will allow stronger causal claims about the cognitive-developmental trajectory of PLEs.

In the context of the large body of previous research, tentative clinical implications may be made. We found associations between receptive language and working memory with PLEs in our younger CUES sample, which supports previous research. This suggests that early intervention to improve these cognitive processes in younger children may be useful at a population level. This could be incorporated into social and emotional well-being curricula via existing mental health promotion programmes in primary schools [47] in collaboration with funders, teachers and parents. For older adolescents seeking help for distressing PLEs, general psychopathology (i.e. total internalising/externalising difficulties) was the strongest predictor of PLE distress severity which is in line with the view of an emotional dysregulation model of distressing PLEs. This possibly supports the use of therapeutic strategies targeting general emotional and behavioural processes which are hypothesised to drive PLE distress [8]. However, in the context of our study limitations, future research in other clinical groups is needed to assess the feasibility of adapting existing interventions for young people reporting distressing PLEs. These prevention and early intervention efforts may have important implications for considering the wider societal consequences of PLEs, particularly given emerging evidence of associations between PLEs, poorer educational achievements [26–29] and later vocational status [30].

## Data Availability

The clinical data for this study are not publicly available, though may be made available from the Principal Investigator upon reasonable request (suzanne.jolley@kcl.ac.uk).
